# UGV-NBWASTE: An oriented dataset for non-biodegradable waste in Bangladesh

**DOI:** 10.1016/j.dib.2025.111559

**Published:** 2025-04-14

**Authors:** Md. Riadul Isalm, Nabil Bin Mahabub, Md. Jubayar Alam Rafi, Pronoy Kanti Roy, Turjo Roy, Md. Tariqul Islam, Md. Abdur Razzak

**Affiliations:** a Department of Computer Science and Engineering, University of Global Village, Barisal 8200, Bangladesh; b Department of Computer Science and Engineering, Daffodil International University, Bangladesh

**Keywords:** Computer vision, Deep learning, Image analysis, Waste detection, Waste classification

## Abstract

The “UGV-NBWASTE” dataset is built for those who manage non-biodegradable waste. The selection of non-biodegradable waste has been decided on adverse environmental conditions, particularly waste management in landfills and water. The dataset is collected from the Barisal district of Bangladesh, where eight distinct types of waste (Plastic Bottle, Hard Plastic, Mask, Medicine Packet, Packet, Polythene, Cocksheet, and Plastic Sandal) are selected based on their widespread use, durability, and difficulty in recycling or managing them via conventional waste disposal methods. Furthermore, waste images are captured using smartphones in indoor and outdoor situations, such as floating in water or partially buried in the mud, which is crucial to diversifying the dataset for effective detection and classification. After data collection, various techniques are applied during the image pre-processing stage to significantly improve the quality of the original images. These include Image Quality Assurance (i.e., image verification and image cleaning) and Image Enhancement (i.e., brightness normalization and image resizing). Then, all images are annotated in oriented bounding box (OBB) format, ensuring waste detection at different angles. The total number of original images is 3600. Waste can be reliably identified whether it is flat, crumpled, or partially obscured, which guarantees the dataset's ability to identify waste in different circumstances and orientations.

Specifications Table

**Table:** 

Subject	Waste Management and Disposal
Specific subject area	Different types of non-biodegradable waste detection and classification
Data Format	Raw image: (.jpg) Annotated text file: (.txt)
Type of data	Image and text file
Data size	Raw images (jpg file): 3600 Annotation (.txt file): 3600
Data collection	The proposed non-biodegradable waste dataset, namely “UGV-NBWASTE,” is collected from the southern part of Bangladesh, particularly the Barisal district. The dataset contains eight types of waste: Plastic Bottle, Hard Plastic, Mask, Medicine Packet, Packet, Polythene, Cocksheet (Styrofoam), and Plastic Sandal. To capture the image the following smartphones are used: iPhone 12 pro max (12 MP 4032 × 3024), Realme 9 speed edition 5 G (48 MP 2412×1080), Samsung Galaxy M21 (48 MP 2340×1080), and Samsung Galaxy s20 Plus (64 MP 3200×1440). After capturing, all the images were manually inspected to ensure they aligned with dataset requirements (e.g., correct object category, clear visibility, and minimal occlusion). Then, the data-cleaning section removed blurry, noisy, and invisible images. Then, contrast stretching was used to normalize brightness variations across images, ensuring consistency in illumination and enhancing feature extraction, and resized to 640×640. Finally, all the images are annotated in the oriented bounding box (OBB) format.
Data source location	This data has been collected from local places in Barisal, Bangladesh. Such as: • Nothullabad Barisal-8200, Bangladesh. • Chowmatha Barisal-8200, Bangladesh. • Rupatoli Barisal-8200, Bangladesh. • B.M College, Barisal −8200, Bangladesh. • Sher-E-Bangla Medical College, Barisal-8200, Bangladesh.
Data accessibility	Repository name: UGV-NBWASTE: An Oriented Non-Biodegradable Waste Dataset in Bangladesh Data identification number: 10.17632/fv28xxn4f3.3 Direct URL to data:https://data.mendeley.com/datasets/fv28xxn4f3/3

## 1 Value of the Data

• The “UGV-NBWASTE” dataset is collected for individuals working in non-biodegradable waste management. As the worldwide waste situation grows worse, handling non-biodegradable materials has become a top priority for environmental professionals, legislators, and researchers.
• The waste images are captured using smartphones in different outdoor situations, such as floating in water or partially buried in the mud, which is crucial to diversifying the dataset for effective detection and recognition.
• The dataset consists of eight distinct categories with a total of 3600 original images.
• All images are annotated in oriented bounding box (OBB) format which ensures the detection of waste at different angles to make it well-suited for real-world applications, including IoT-based waste management.
• This dataset can be used by researchers to assess and compare model performance for tasks involving waste detection and classification in practical settings. The dataset is especially well-suited for researching difficult identification issues in non-biodegradable waste management because of its diversity and orientation annotations.

## 2 Background

Non-biodegradable waste is the particles that are not biodegradable naturally and remain unchanged in the environment indefinitely. As time passes, this waste breaks down into tiny pieces known as microplastics, which are unable to dissolve and remain in the environment forever, damaging the food chain and causing health risks to humans [1] . Several well-known datasets have been proposed in this area [1,2]. Wahidur et.al. [3] Introduced a dataset called “BDWaste,” which classified Bangladeshi waste into two categories: biodegradable and non-biodegradable. The dataset was built for waste classification and collected in both indoor and outdoor environments with plain backgrounds. To make a dataset more diverse (e.g., floating in water, partially submerged in soil), it is essential to ensure data in the outdoor environment for both detection (e.g., using YOLOv8) and recognition [4,5] . Recently, Pronay et.al [6]. Introduced a non-biodegradable plastic waste detection and classification (NPWDC) method. Research on waste detection has greatly benefited from existing datasets, yet there are still gaps in environmental diversity, waste classification, and practical applicability. Table 1 summarizes key datasets in this domain. Existing datasets such as TrashNet [7] and Garbage Classification [8] only apply to the classification of waste indoors with controlled backgrounds, whereas FloW [5] and BePLi Dataset v1 [4] concentrate on locations, including beaches and inland waters, and have limited generalizability. Larger databases, such as WasteNet [9]and TACO [10], are more varied, although they concentrate on general litter rather than environmental complexity or non-biodegradable garbage.

**Table 1:** Comparative overview of related datasets in waste detection and classification.

Dataset name	Images	Application	Annotation	Cate- gories	Key contributions
Garbage Classification [8]	15,150	Classification	Directory-Based	12	Focuses on 12 classes of household garbage for classification tasks.
WasteNet [9]	15,000	Classification	Directory-Based	30	General-purpose waste detection and classification dataset.
TrashNet [7]	2527	Classification	Directory-Based	6	Dataset with images of six different waste categories for classification tasks.
BDWaste [3]	2497	Classification	Directory-Based	20	Focuses on detecting and classifying digestible and indigestible waste types in Bangladesh.
TACO [10]	5700	Detection and Segmentation	Cropped annotated	11	Provides diverse litter images for waste detection in outdoor environments.
BePLi Dataset v1 [4]	3709	Detection and Segmentation	Bounding Box and Pixel-Based	5	For instance, for the segmentation of beach plastic litter.
FloW [5]	2000	Detection and Classification	Bounding Box	1	Detects floating waste in aquatic environments for environmental monitoring.
**UGV-NBWASTE**	3600	Detection and Classification	Oriented Bounding Box	8	Focuses on detecting and classifying non-biodegradable waste materials captured in diverse environments

The most common large-scale datasets are Garbage Classification, WasteNet, TrashNet, and BDWaste [3,7,8,9]. Although these datasets contain significant data, they mainly focus on classification tasks. In contrast, these dataset follows a directory-based annotation structure. Additionally, most existing datasets have simple backgrounds. Furthermore, these datasets include both biodegradable and non-biodegradable waste, but the diversity of non-biodegradable waste is limited. In contrast, the TACO, BePLi Dataset v1, and FloW datasets use cropped, bounding-box, and pixel-based annotations, which make them suitable for detection, segmentation, and classification [4,5,10]. Although these datasets include plastic and other waste categories, the diversity of non-biodegradable waste types is still limited, and some categories are irrelevant for Bangladesh. Our goal is to collect non-biodegradable waste data for Bangladesh, especially for the southern region. While other open-source datasets are comprehensive, they are not always suitable for the Bangladeshi context. The BDWaste dataset is consistent with the Bangladeshi perspective, it is not designed for object Detection.

However, the proposed UGV-NBWASTE disapproves a tailored dataset exclusively for non-biodegradable trash, such as Plastic Bottle, Hard Plastic, Mask, Medicine Packet, Packet, Polythene, Cocksheet (Styrofoam), and Plastic sandals. 3600 images taken in various real-world locations, including soil, water, and urban areas. Additionally, it employs oriented bounding boxes, making it highly suitable for waste detection and classification in practical scenarios.

## 3 Data Description

The proposed “UGV-NBWASTE” dataset focuses on non-biodegradable waste, which is collected from Barisal, Bangladesh (since Bangladesh is a small country, the waste in Barisal is representative of waste across the nation). Table 2 provides a brief description of the waste sample images, along with the number of collected original images and their local names. The dataset consists of eight distinct classes (i.e., Plastic Bottle, Hard Plastic, Mask, Medicine Packet, Packet, Polythene, Cocksheet, and Plastic Sandal), each class containing 450 original images, a total of 3600 images (=8 × 450).

**Table 2:** “UGV-NBWASTE” dataset description table.

No.	Name	Quantity	description	Image
1	Plastic Bottle	450	Plastic bottles are portable, durable, cheap, and flexible. They can hold drinks such as water, energy drinks, and juice. The size ranges from 250 ml to 5 liters and consists of four parts: the body, the neck, the cap, and the base.	
2	Hard Plastic	450	Hard plastic is a sturdy and stiff material used in various items owing to its strength and dependability. The polymerization of monomers creates hard plastic.	
3	Mask	450	Masks are protective coverings used to cover the mouth and nose to prevent the possible inhalation of harmful particles or pollutants. Disposable masks are made from Polypropylene, which is a type of plastic.	
4	Medicine Packet	450	A silver (mostly) colored plastic packets used for medicine preservation. The upper layer consists of a plastic sheet with cavities to hold the pills and the back layer is sealed with foil backing.	
5	Packet	450	Packets refer to small but compact plastic used to contain, protect, or transport various items like food, and general goods.	
6	Polythene	450	Polythene is a thin, soft, and bendy plastic material, which is widely used all over the world. It is made from Polymerization of Ethylene. Due to its lightweight and water-resistance capability, it is commonly used to carry goods or other materials.	
7	Cocksheet	450	A cocksheet (Styrofoam) is a type of synthetic or plastic sheet mostly used in the packaging, transportation, and construction industries. It is made from substances like PVC (Polyvinyl Chloride), making them really durable.	
8	Plastic Sandal	450	Plastic sandals are widely used because they are lightweight, flexible, affordable, and durable. Made from materials such as PVC or polyethylene.	
	Total:	3600		

**Fig. 1:**
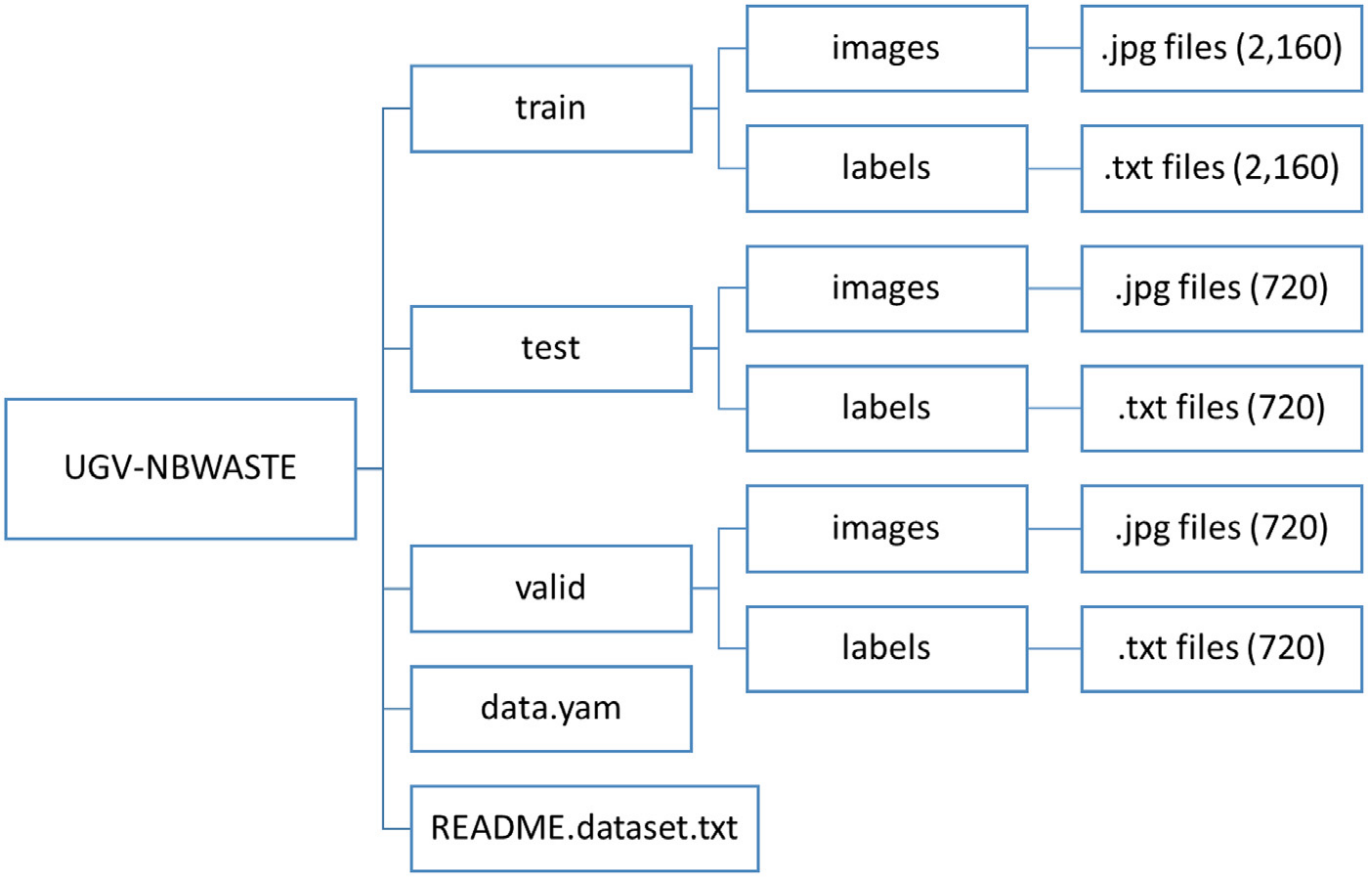
Directory and subdirectory structure and files of the “UGV-NBWASTE” dataset.

Fig. 1 illustrates the directories and files of the proposed dataset. The root directory named *“UGV - NBWASTE”* includes three sub-directories (i.e., train, test, and valid) and two files (i.e., data.yam and README.dataset.txt. Each of the three sub-directories contains two additional sub-directories (images and labels). The original image files (e.g., 1001.jpg) are stored in the images directories, while the corresponding annotated files (e.g., 1001.txt) are stored in the labels directories. This annotation file uses the OBB format (i.e.,class_ID,x1,y1,x2,y2,x3,y3,x4,y4) to identify and classify trash. The class_ID denotes the trash kind (i.e., polythene=0, packets=1, bottle=2, hard plastic=3, gloves=4, medication packets=5, cocksheet=6, plastic sandal=7). Each bounding box includes four corner points: (x1,y1) for the first,(x2,y2)for the second, (x3,y3)for the third, and (x4,y4) for the fourth, which ensures that the item is correctly oriented and positioned. The “*data.yam*” file specifies the dataset's folder directory, the total number of classes, and class names. The dataset is annotated in the OBB format, which is compatible with YOLOv8. However, the RoboFlow Data Vision link provided in the “*README.dataset.txt*” file can generate popular data formats, including all YOLO versions, COCO JSON, TFRecord, Pascal VOC XML, etc. Finally, three sub-directories (i.e., train, test, and validate) represent 3600 images spited in a ratio of (3:1:1) where 2160 images (60 %) are for training, 720 images (20 %) are for validation, and 720 images (20 %) are for testing.

## 4 Experimental Design, Materials, and Methods

**Fig. 2:**
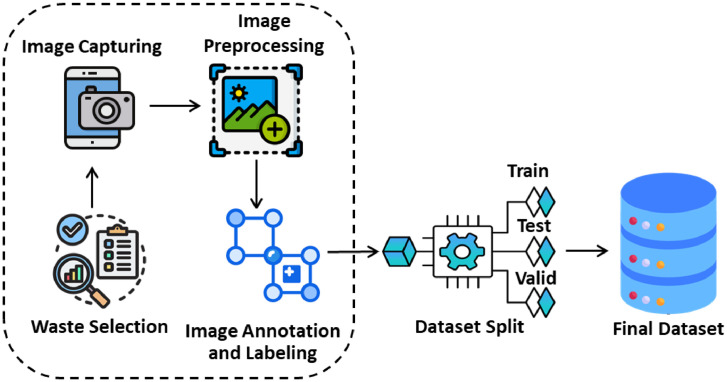
General architecture of the “UGV-NBWASTE” dataset.

Fig. 2 illustrates the general architecture of the proposed 'UGV-NBWASTE' dataset, which mentions fundamental phases, including waste selection, waste capturing, image preprocessing, image annotation and labeling, and data siltation, which are necessary for improving and preparing the dataset. Initially, various non-biodegradable waste items are selected (i.e., Plastic Bottle, Hard Plastic, Mask, Medicine Packet, Packet, Polythene, Cocksheet, and Plastic Sandal) from the southern part of Bangladesh, and high-resolution cameras are used to capture the waste under different circumstances. After quality-enhancing preprocessing, the images are thoroughly annotated and labeled using the Roboflow platform [11]. Finally, the dataset is divided into testing, validation, and training sets.

### 4.1 Waste selection

Waste selection is the initial phase of selecting different non-biodegradable waste materials. The proposed “UGV-NBWAST” dataset selects eight distinct types of waste (i.e., Plastic Bottle, Hard Plastic, Mask, Medicine Packet, Packet, Polythene, Cocksheet, and Plastic Sandal) based on their widespread use, durability, and difficulty in recycling or managing them via conventional waste disposal methods, which is discussed in more detail in “DATA DESCRIPTION” Section.

### 4.2 Image capturing

The UGV-NBWASTE collection comprises distinct waste entities as well as several images of the same waste taken in various settings (e.g., partial occlusion, different lighting, or different angles). A single subset (training, validation, or test) was given enriched versions and various views of the same waste entity during data splitting to preserve dataset integrity and prevent data leaking. Table 3 shows the configuration details of the devices used during data collection, including device names, detailed camera specifications, and resolutions. The use of multiple devices ensures diversity in image quality, accounting for variations in perspective, color accuracy, and lighting conditions [3]. To make the dataset more adaptable to practical applications where images may be captured in a variety of situations, such as different light conditions, different backgrounds, and from different angles, which are shown in Table 4, Table 5. Table 4 provides a summary of the data acquisition process, outlining the environmental conditions and collection methods applied to the dataset. This significantly improves the quality of the dataset, ensuring that models trained on the dataset will adapt better to different settings. Table 5 provides a structured overview of the different camera angles, viewing angles, and distances used during data collection. Different angles help the models detect objects in multiple orientations, while different distances account for shape perception and different depths. This structured approach increases the robustness of the dataset.

**Table 3:** Device name and configuration details.

Device name	Camera specifications	Resolution
iPhone 12 Pro Max	12 MP, f/1.6, 26 mm (wide), 1.7 µm, dual pixel PDAF, sensor-shift OIS; 12 MP, f/2.2, 65 mm (telephoto), PDAF, OIS, 2.5x optical zoom; 12 MP, f/2.4, 120°, 13 mm (ultrawide)	4032 × 3024 (12 MP)
Samsung Galaxy M21	48 MP, f/2.0, 26 mm (wide), PDAF; 8 MP, f/2.2, 123° (ultrawide); 5 MP, f/2.2 (depth)	8000 × 6000 (48 MP)
Samsung Galaxy S20 Plus	64 MP, f/2.0, 29 mm (telephoto), PDAF, OIS, 1.1x optical zoom; 12 MP, f/1.8, 26 mm (wide), 1.8 µm, dual pixel PDAF, OIS; 12 MP, f/2.2, 120° (ultrawide)	9216 × 6912 (64 MP)
Realme 9 Speed Edition 5G	48 MP, f/1.8, (wide), PDAF; 2 MP, f/2.4 (depth); 2 MP, f/2.4 (macro)	8000 × 6000 (48 MP)

**Table 4:** Data acquisition table.

Parameter	Details
Environment	Indoor and Outdoor
Background	Plain background (indoor) and natural and varied backgrounds (outdoor)
Captured By	Four Person
Lighting	Natural light (Sunny, cloudy) and artificial light

**Table 5:** **R**elation between view & angle and distance for selected items.

View	Approx. Angle	Selected Items	Distance (cm/m)	Example
Top View	90° (Overhead)	Small flat items (Mask, Medicine Packet, Packet, Polythene)	30–80 cm	
Side View	45°–60°	3D objects (Plastic Bottle, Hard Plastic, Cocksheet)	30–100 cm	
Front View	0°–30°	Larger plastic objects (Hard Plastic, Plastic Bottle)	50 cm–1 m	
Wide View	10°–30°	Grouped objects or arranged dataset items	1–3 m	

**Fig. 3:**
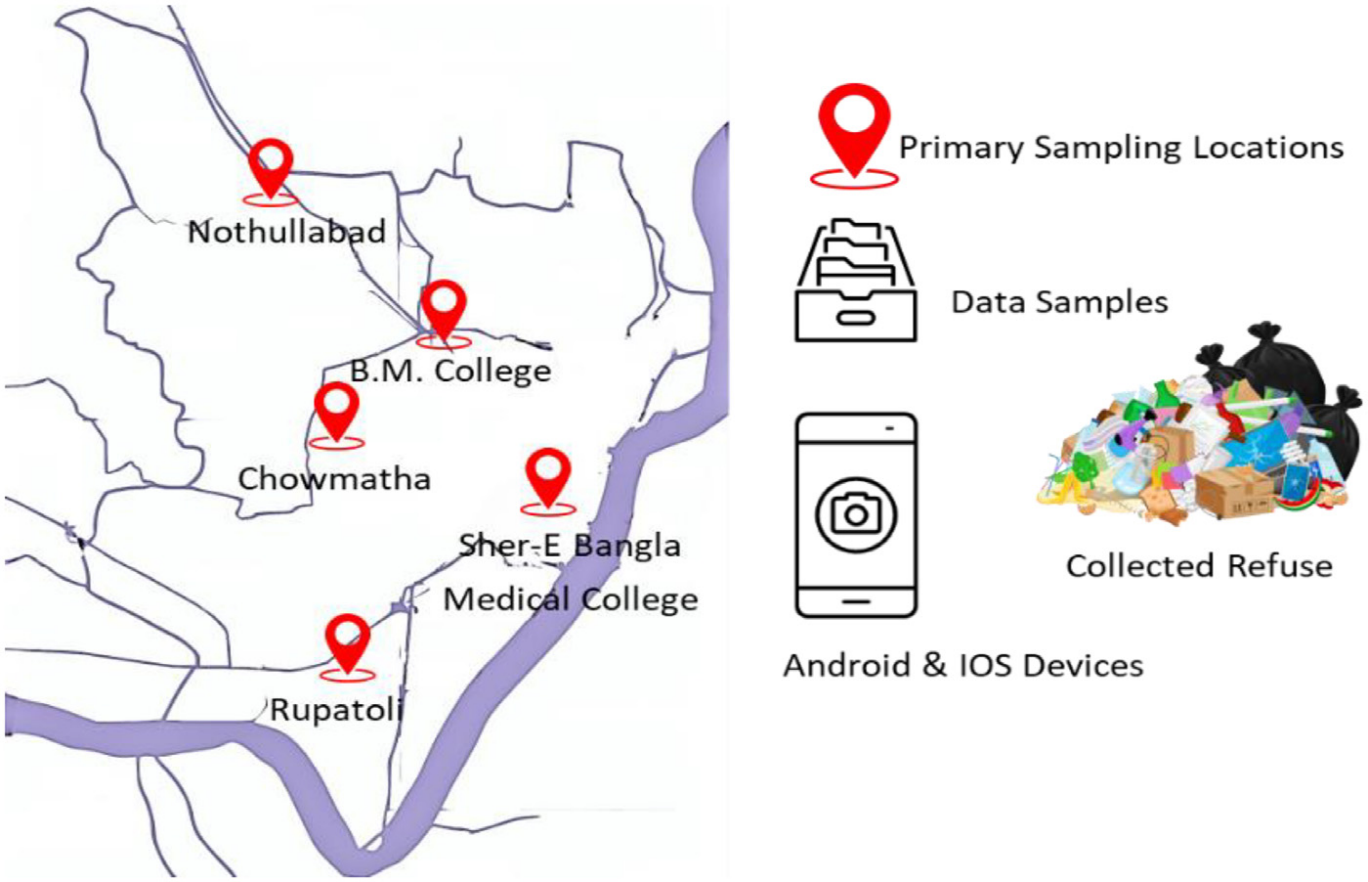
Map view of collected images from Barisal city, Bangladesh.

The primary location for collecting data is the southern part of Bangladesh, particularly the Barisal district. Fig. 3 illustrates the Key locations in this area, as in Nothullabad, B.M. College, Chowmatha, Sher-E Bangla Medical College, and Rupatoli. These sites are strategically chosen to ensure a comprehensive collection of waste types from various urban settings within the region.

**Fig. 4:**
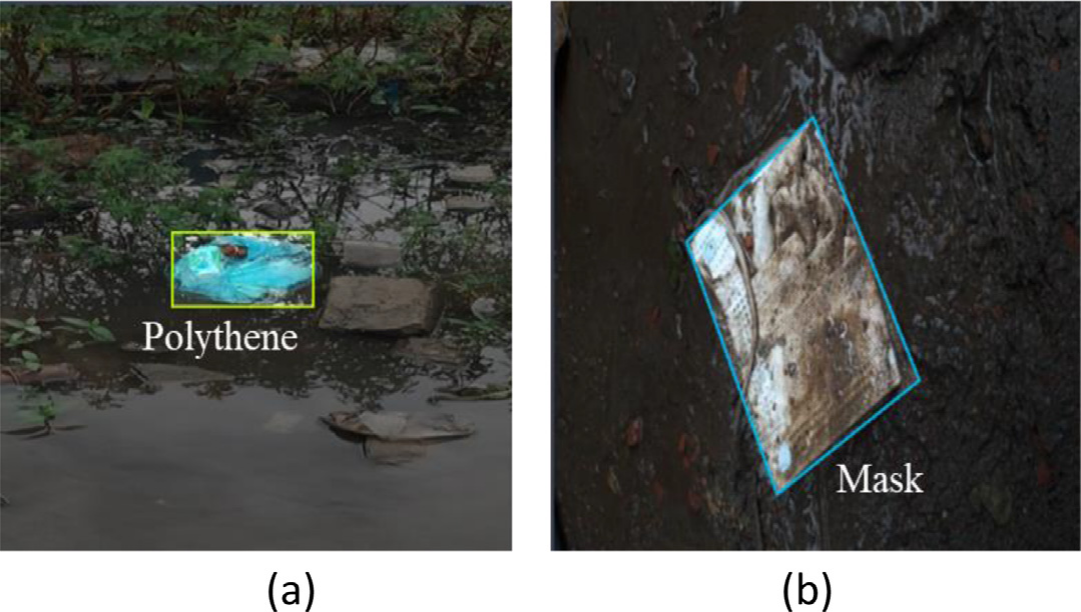
Example of Data collection diversity. (a) Waste floating in the water; (b) Waste partially submerged in soil.

Fig. 4 depicts the diversity of the dataset, which is enhanced by capturing waste in various conditions:

• **Waste Floating in Water:** Images of waste floating in water bodies to understand how waste interacts with aquatic environments.
• **Waste Partially Submerged in Soil:** Capturing waste that is partially buried or submerged in soil, highlighting decomposition stages and potential soil contamination.

This multi-faceted approach to data collection not only enriches the dataset but also provides a comprehensive view of waste management challenges in urban and semi-urban settings of southern Bangladesh. All waste is collected from both indoor and outdoor environments (e.g., along the roadside, parks & public places, outdoor shops, and markets). Fig. 4, Fig. 4 illustrate the examples of diver’s data collection such as, waste floating in the water, waste partially submerged in soil, and multiple classes of waste in the same image. After the data collection, to organize them efficiently each piece of data was organized in a storage drive into separate folders according to their class names.

### 4.3 Image pre-processing

Various techniques are applied during the image pre-processing stage to significantly improve the quality of the original images. These include Image Quality Assurance (i.e., image verification and image cleaning) and Image Enhancement (i.e., brightness normalization and image resizing). The process of image quality assurance is as follows:

• **Image Validation:** After capturing, all the images were manually inspected whether images meet certain criteria before using them in a dataset such as, ensures that images belong to the correct object category, are formatted correctly, are not corrupted, meet acceptable resolution standards, have correct labels, are free of duplicates and do not contain multiple objects in a single image. Each image should belong to only one class for clear labeling and to avoid confusion in training models.
• **Image cleaning:** To improve the quality of datasets by ensuring clear visibility, reducing clutter, and removing blurry, noisy, or invisible images.

**Fig. 5:**
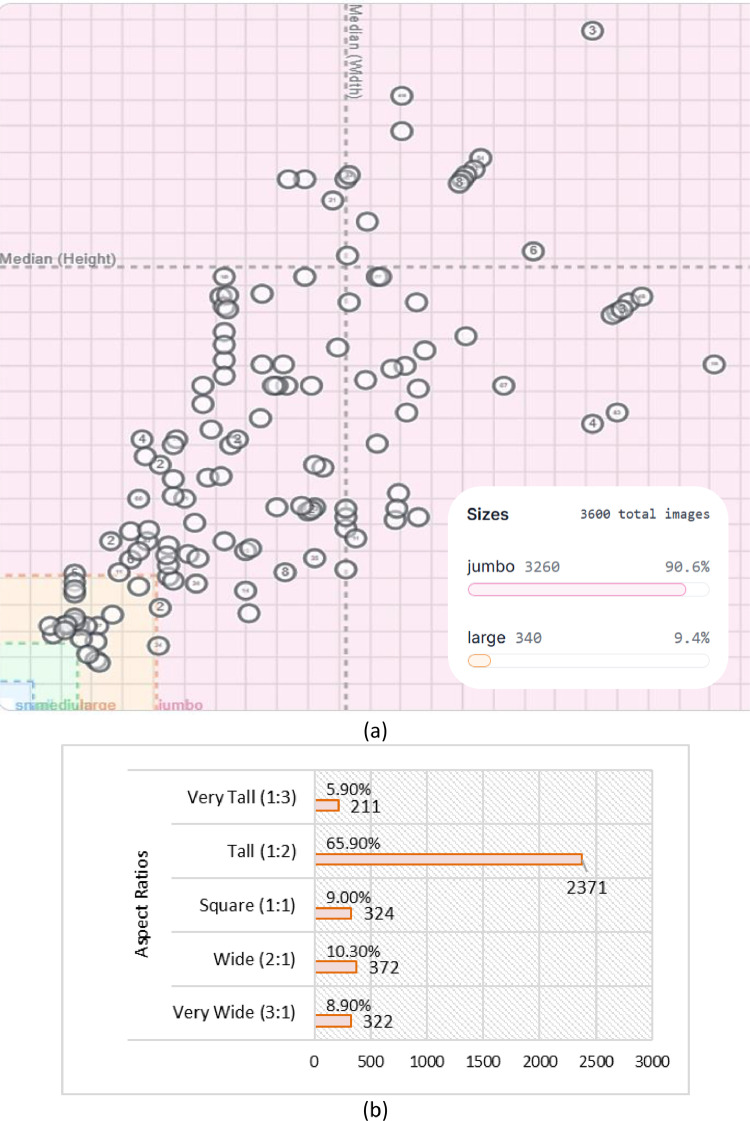
Dimension Insights (a) The distribution of original image dimension. Image sizes, jumbo (maximum 10,240 px) images 3260 and large (i.e., maximum 1024 px) images 340.; (b) Aspect Ratio of original image such as vary tall (1:3), tall (1:2), square (1:1), wide (2:1) and vary wide (3:1).

After completing image verification and cleaning, a total of 3600 (=8 × 450) original images remained, with an average size of 9.00 MP and an average resolution of 2250 × 3425. Fig. 5(a) and (b) explain the distribution of the original image dimension within the dataset.

The dashed lines in Fig. 5(a) indicate the median width of 2250 pixels and the height of 3425 pixels, which are the dimensions of the reference image in the dataset. Fig. 5(b) shows how the images in the dataset vary in size and aspect ratio, such as vary tall (1:3), tall (1:2), square (1:1), wide (2:1), and vary wide (3:1). While, the datasets are collected from smartphones, which is why 65.90 % of the images are mostly tall. After this, the image enhancement process is applied as follows:

• **Normalized brightness:** Then, contrast stretching was used to normalize brightness variations across images, ensuring consistency in illumination and enhancing feature extraction.
• **Resized Image:** Finally, each image is resized to a uniform dimension of 640×640 pixels.

### 4.4 Image annotation and labelling

**Fig. 6:**
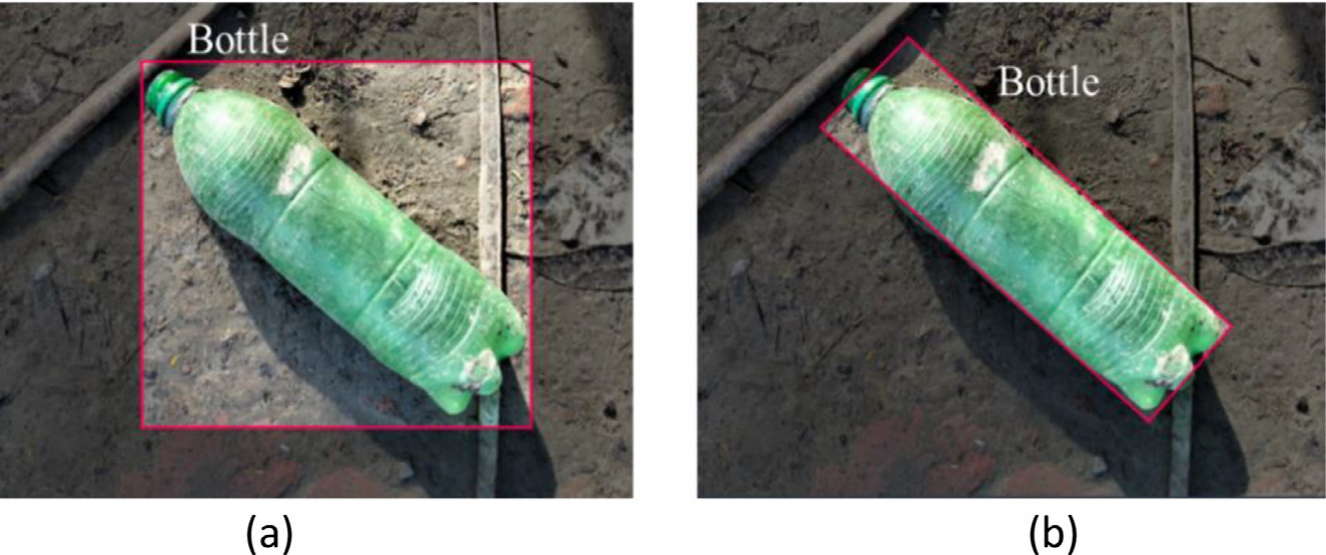
Sample image annotation (a) Unoriented bounded box annotation; (b) Oriented bounding box annotation.

**Fig. 7:**
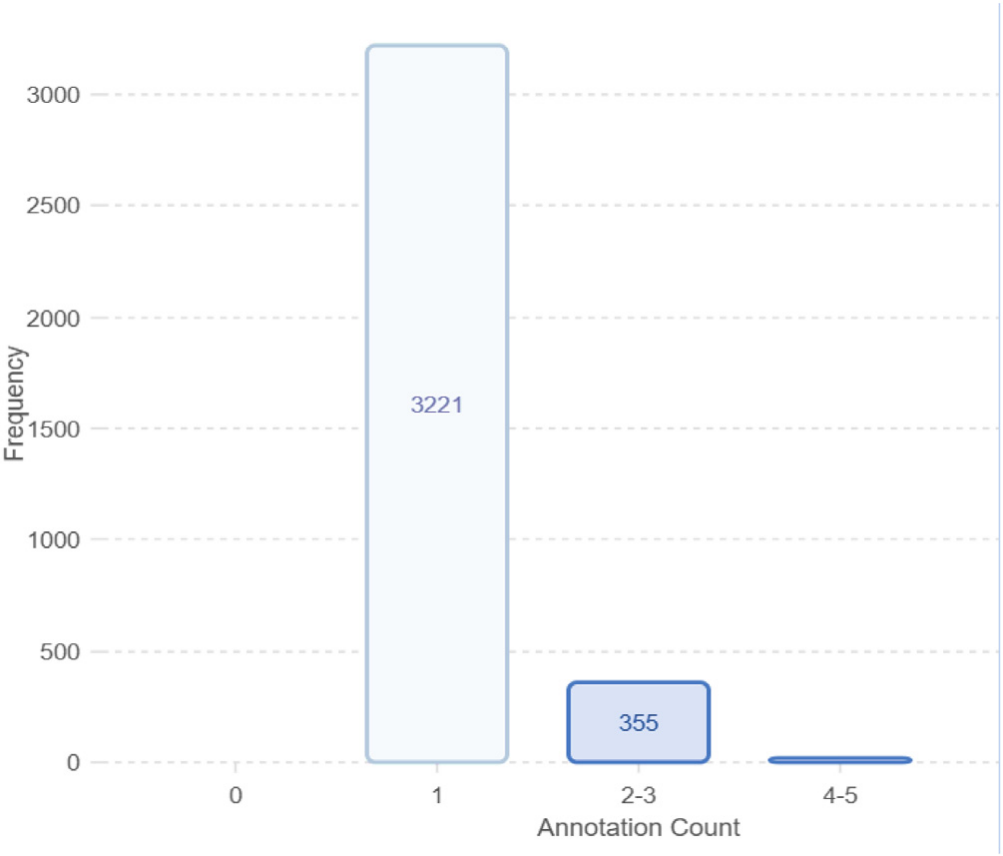
Overviews of frequency annotations in each image in the dataset.

After completing the pre-processing phase all 3600 images are manually annotated, and objects within the image were carefully marked to ensure precise identification which is shown in Fig.6. Each image is then assigned to its corresponding classes: Plastic Bottle, Hard Plastic, Mask, Medicine Packet, Packet, Polythene, Cocksheet and Plastic Sandals. Fig. 6(b) shows the objects are annotated according to their orientation, whether flat or partially obscured. This carefully OBB-annotated approach allows the model to detect waste materials effectively, regardless of their position or condition, such as crumpled packets or tilted bottles, as demonstrated in Fig. 6(a) and (b). Following annotation and classification, the images were also resized to appropriate dimensions for pre-processing. Fig. 7 provides a summary of the annotations across all images in the dataset. As shown in Table 6, there are a total of 4095 annotations for 3600 images, with an average of 1.1 annotations per image.

**Table 6:** Annotations data description.

Number of images	Number of annotations	Average annotations
3600	4095	1.1 per image

### 4.5 Dataset splits

**Fig. 8:**
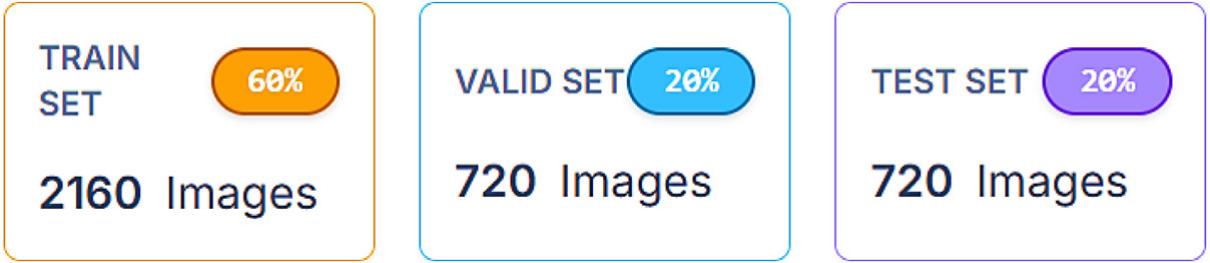
Dataset Split in a ratio of (3:1:1).

The dataset consists of 3600 original images. To ensure proportionate representation of all waste categories, the dataset was divided in a ratio of (3:1:1) where 2160 images (60 %) are for training, 720 images (20 %) are for validation, and 720 images (20 %) are for testing using a random but stratified method depict in Fig. 8. Additionally, the RoboFlow data vision link is provided in the “*README.dataset.txt*” file, where data splits can be customized as needed.

## Limitations

The dataset “UGV-NBWASTE” has some drawbacks in terms of both data collection and evaluation. First, the data was exclusively collected from the Barisal Region, while Bangladesh is dealing with a major waste problem. Following this, although there are many different types of waste found in real-world situations, this dataset contains only eight distinct waste types. Potentially limiting the representation of broader waste diversity. Also, it is crucial to understand that waste categories can vary substantially worldwide. However, the data only focuses on one waste category: non-biodegradable.

## Ethics Statement

The authors attest that their work and the data set they have supplied fully satisfy the ethical standards for publishing in Data in Brief, as stated in “https://www.elsevier.com/authors/journal-authors/policies-and-ethics. To publish in Data in Brief and confirm that the current work does not involve human subjects, animal experiments, or any data collected from social media platforms.

## CRediT Author Statement

**Md. Riadul Isalm:** Conceptualization, Data annotation, Methodology, Investigation, Writing - Review & Editing, Visualization, Supervision. **Nabil Bin Mahabub:** Data collection, Data annotation, Software, Formal analysis **Md. Jubayar Alam Rafi:** Data collection, Writing - Original Draft, Visualization. **Pronoy Kanti Roy**: Data collection, Conceptualization, Methodology, Software; **Turjo Roy:** Data collection, Writing - Original Draft, Visualization. **Md. Tariqul Islam:** Conceptualization, Methodology. **Abdur Razzak:** Validation, Formal analysis.

## Declaration of Generative AI and AI-Assisted Technologies in the Writing Process

During the preparation of this work, we used **Grammarly, Google Translator, and Quillbot** to improve the writing process, readability, and language of the work. After using this tool/service, we reviewed and edited the content as needed and take(s) full responsibility for the content of the publication.

## Declaration of Competing Interest

The authors declare that they have no known competing financial interests or personal relationships that could have appeared to influence the work reported in this paper.

## Data Availability

Mendeley DataUGV-NBWASTE: An Oriented Non-Biodegradable Waste Dataset in Bangladesh (Original data)
